# Parity influences on the infant gut microbiome development: a longitudinal cohort study

**DOI:** 10.1080/19490976.2025.2557980

**Published:** 2025-09-09

**Authors:** R. Prasad, A. Angelova, P. Subramanian, S. Namasivayam, Q. Chen, K. Banks, S. Levy, G. L. Maxwell, S. K. Hourigan

**Affiliations:** a Clinical Microbiome Unit, Laboratory of Host Immunity and Microbiome, Division of Intramural Research, National Institute of Allergy and Infectious Disease, National Institute of Health, Bethesda, MD, USA; b Bioinformatics and Computational Biosciences Branch, National Institute of Allergy and Infectious Disease, National Institute of Health, Bethesda, MD, USA; c Women’s Service Line, Inova Health System, Falls Church, VA, USA

**Keywords:** Microbiota, pregnancy, neonate, delivery mode, peripartum antibiotics

## Abstract

Parity, the number of pregnancies carried beyond 20 weeks, influences the maternal gut microbiome. However, whether parity modulates the infant microbiome longitudinally remains underexplored. To address this, 746 infants in a longitudinal cohort study were assessed. Serial infant stool samples collected at 2, 6, 12, and 24 months underwent 16S ribosomal RNA gene sequencing. Mothers were stratified by parity: 1 = 32.6%, 2 = 42.0%, 3 = 18.6%, 4, 5, 6 or 7 = 6.8%. Although no differences in alpha diversity were found with parity, significant differences were found in microbiome composition (beta diversity, Bray-Curtis) by parity at each time point through the first year of life (*p* < 0.001). Delivery mode (vaginal delivery (VD) = 60.5%, Cesarean section (CS) = 39.5%) was a significant contributor to infant microbiome composition at 2 months (*p* = 0.002). In VD infants, parity-related differences in microbiota composition were evident up to 6 months (*p* ≤ 0.002), however in CS delivered infants, early life parity-related differences were absent. In conclusion, our brief report showed a significant effect of parity on early infant gut microbiome composition. However, the effect of parity diminished with CS delivery, which we hypothesize is due to decreased mother-to-infant microbiome transfer with CS. These results demonstrate the necessity of including parity in longitudinal infant microbiome analyses.

## GRAPHICAL ABSTRACT

**Figure:**
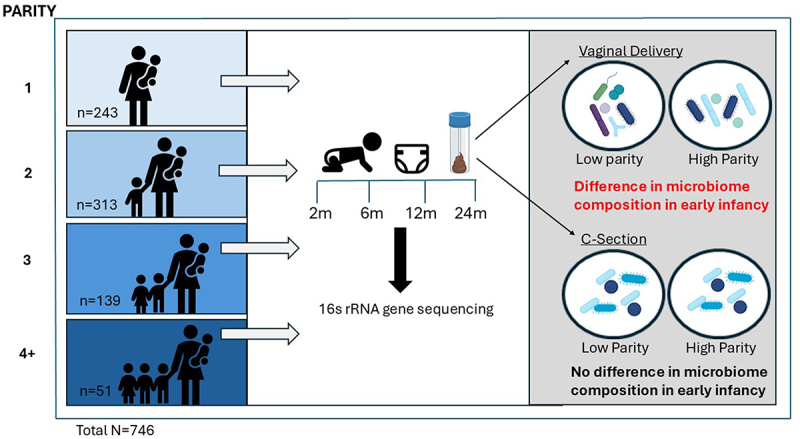
Schematic figure of study. Parity influences the infant gut microbiome composition in early life, with parity effects diminished in CS.

## Introduction

Emerging research increasingly highlights the significant role of the maternal gut microbiome during pregnancy, not only in maintaining maternal health but also in influencing the longitudinal health of the infant.^1–3^ While various maternal factors influencing the maternal and infant gut microbiome composition have been identified, such as delivery mode, peripartum antibiotics exposure and maternal pre-pregnancy BMI, additional contributing factors are not fully understood.^4–8^ An area that remains underexplored is the potential impact of maternal parity, the number of pregnancies carried beyond 20 weeks, on infant microbiome dynamics.

Animal models have shown that parity can influence the maternal and infant gut microbiome composition. In swine models, maternal microbial composition showed an increased rate of change over the course of gestation in low-parity animals, while high-parity animals exhibited a reduction in this change.^9^ In the piglets, higher bacterial diversity was found in those born to primiparous sows compared with multiparous sows, with differentially abundant taxa between the two groups over the first few weeks of life.^9,10^

In humans, Kennedy et al. showed in a prospective small cohort study (*n* = 65) that primiparous women with higher pre-pregnancy body mass index (BMI) exhibited fewer changes in their gut microbiota throughout pregnancy and six-month postpartum compared to those with lower pre-pregnancy BMI^11^. However, in multiparous women, this pattern of microbiome alteration was not observed, suggesting that the first pregnancy may trigger unique microbiome adaptations that persist and potentially influence subsequent pregnancies. This effect extended to the infant microbiome, as the effects of gestational weight gain (GWG) and perinatal antibiotic exposure on the infant microbiome composition of infants at 6 months of age were modulated by parity.^11^ A significant effect of perinatal antibiotics on infant microbiome composition at 6 months was only found in infants of primiparous women but not multiparous women.^11^

Extending these findings, Kennedy et al. then examined meconium samples between primiparous and multiparous infants who were vaginally delivered and without peripartum antibiotic exposure.^12^ They showed that although birth order did not significantly impact either alpha or beta diversity, the mean relative abundance of *Bifidobacterium sp* was greater in neonates of multiparous participants compared to those of primiparous participants.^12^ Additionally, a much larger study from Finland comparing the microbiome of infants delivered to primiparous versus multiparous women demonstrated that parity significantly contributed to the developing infant microbiome to different degrees over the first 2 years of life.^13^

Given the findings from these studies and the importance of the developing infant microbiome in setting the stage for immune and metabolic regulation, there is a need for further research examining parity’s effects on the developing infant microbiome n.^14,15^ To build on the current knowledge, in this brief report we investigated the effects of parity on the infant gut microbiome longitudinally in a large cohort study (*n* = 746 infants) up to 2 years of age, examining individual parity groups (parity of 1, 2, 3, 4, etc.) and associated factors.

## Results

### Subjects, demographics, and clinical characteristics

A total of 746 mother-infant dyads were included in this analysis (stool samples are only available for infants). Demographic and clinical data for infants and their mothers are shown in Table 1. Among mothers, 32.6% had a parity of 1, 42.0% had a parity of 2, 18.6% had a parity of 3, 5.1% had a parity 4, 1.1% had a parity of 5, 0.5% had a parity 6 and 0.1% had a parity of 7. Because of the low numbers for parities 5 through 7, parities 4,5, 6, or 7 were combined for further analyses (6.8%) (Table 1). Clinical and demographic variables were evaluated for any significant associations.

**Table 1.** Distribution of clinical and demographic variables distributed by parity for 746 maternal infant dyads from 2 m, 6 m, 12 m, and 24 m. Bolded p-values are statistically significant. BMI (body mass index). ACOG (American College of obstetricians and gynecologists). Unknown data were not included in the assessment of statistical significance.

Maternal Parity						
	1 (n=243)	2 (n=313)	3 (n=139)	4+ (n=51)		p-value
**Maternal Characteristics**						
**Maternal Ethnicity**						**<0.0001**
Hispanic or Latino	33 (14%)	72 (23%)	53 (38%)		25 (49%)	
Non-Hispanic or Latino	209 (86%)	241 (77%)	86 (62%)		26 (51%)	
Declined to answer/Unknown	1 (0%)	0 (0%)	0 (0%)		0 (0%)	
**Maternal Race**						**0.0042**
Caucasian	161 (66%)	222 (71%)	105 (76%)		34 (67%)	
Asian	44 (18%)	34 (11%)	11 (8%)		2 (4%)	
African American or Black	14 (6%)	11 (4%)	4 (3%)		2 (4%)	
More than one Race	9 (4%)	17 (5%)	4 (3%)		2 (4%)	
Other	15 (6%)	29 (9%)	15 (11%)		11 (22%)	
**Maternal age in years (mean, range)**	32.0 (19–44)	33.1 (19–44)	34.3 (33–41)		33.2 (25–46)	**<0.0001**
**Maternal pre-pregnancy weight in lbs (mean, range)**	145.6 (90–327)	147 (90–250)	148.5 (105–260)		158.6 (115–255)	0.0527
**Maternal pre-pregnancy BMI (kg/m2)**	24.6 (16.4–54.4)	25.1 (15.2–45.7)	25.9 (18.2–40.1)		27.8 (18.4–40.2)	**0.0002**
**Maternal weight gain in pregnancy by ACOG recommendations**						0.3873
Below	38 (16%)	46 (15%)	31 (22%)		8 (16%)	
Within	91 (37%)	104 (33%)	41 (29%)		16 (31%)	
Above	114 (47%)	163 (52%)	67 (48%)		27 (53%)	
**Mode of Delivery**						0.3115
Vaginal	158 (65%)	179 (57%)	84 (60%)		30 (59%)	
Cesarean Section	85 (35%)	134 (43%)	55 (40%)		21 (41%)	
**Prenatal Antibiotics**						0.8705
Yes	51 (21%)	72 (23%)	28 (20%)		10 (20%)	
No	192 (79%)	241 (77%)	111 (80%)		41 (80%)	
**Peripartum Antibiotics**						0.4668
Yes	128 (53%)	184 (59%)	77 (55%)		31 (61%)	
No	115 (47%)	129 (41%)	62 (45%)		20 (39%)	
**Household Income**						**<0.0001**
Less than $49,999	27 (11%)	55 (18%)	43 (31%)		25 (49%)	
$50,000–$99,999	23 (9%)	23 (7%)	11 (8%)		9 (18%)	
$100,000 - $149,999	49 (20%)	52 (17%)	22 (16%)		3 (6%)	
$150,000 - $199,999	50 (21%)	50 (16%)	17 (12%)		1 (2%)	
Greater than $200,000	76 (31%)	109 (35%)	37 (27%)		12 (24%)	
Decline to answer/Unknown	18 (7%)	24 (8%)	9 (6%)		1 (2%)	
**Education**						**<0.0001**
High School	17 (7%)	40 (13%)	38 (27%)		24 (47%)	
Some College/Associate’s degree	12 (5%)	21 (7%)	15 (11%)		8 (16%)	
Bachelor’s Degree	97 (40%)	105 (34%)	33 (24%)		9 (18%)	
Master’s Degree	79 (33%)	107 (34%)	37 (27%)		7 (14%)	
Doctorate/Higher Level of Education	30 (12%)	28 (9%)	14 (10%)		3 (6%)	
Decline to answer/Unknown	8 (3%)	12 (4%)	2 (1%)		0 (0%)	
**Infant Characteristics**						
**Infant Sex**						0.6841
Male	119 (49%)	168 (54%)	69 (50%)		26 (51%)	
Female	124 (51%)	144 (46%)	70 (50%)		25 (49%)	
Unknown	0 (0%)	1 (0%)	0 (0%)		0 (0%)	
**Full term gestational age (≥37 weeks)**						**0.0282**
Yes	220 (91%)	294 (94%)	123 (88%)		42 (82%)	
No	23 (9%)	19 (6%)	16 (12%)		9 (18%)	
**Gestational age in weeks (mean, range)**	38.5 (24–42)	38.6 (28.41)	38.3 (30–41)		37.9 (29–40)	0.0559
**Infant Antibiotic use at birth**						**0.0075**
Yes	13 (5%)	4 (1%)	3 (2%)		4 (8%)	
No	230 (95%)	309 (99%)	136 (98%)		47 (92%)	
**Infant Antibiotic use between birth and 2m**						**0.0232**
Yes	2 (1%)	12 (4%)	7 (5%)		2 (4%)	
No	185 (76%)	228 (73%)	91 (65%)		30 (59%)	
Unknown	56 (23%)	73 (23%)	41 (29%)		19 (37%)	
**Infant Antibiotic use between 2m and 6m**						**0.0310**
Yes	9 (4%)	25 (8%)	11 (8%)		0 (0%)	
No	175 (72%)	204 (65%)	93 (67%)		35 (69%)	
Unknown	59 (24%)	84 (27%)	35 (25%)		16 (31%)	
**Infant Antibiotic use between 6m and 12m**						0.8259
Yes	34 (14%)	42 (13%)	19 (14%)		4 (8%)	
No	148 (61%)	182 (58%)	83 (60%)		29 (57%)	
Unknown	61 (25%)	89 (28%)	37 (27%)		18 (35%)	
**Infant Antibiotic use between 12m and 24m**						0.1141
Yes	11 (5%)	25 (8%)	6 (4%)		1 (2%)	
No	123 (51%)	133 (42%)	64 (46%)		20 (39%)	
Unknown	109 (45%)	155 (50%)	69 (50%)		30 (59%)	
**Breastmilk/Formula at 6m**						0.1168
Exclusive Breastmilk	70 (29%)	97 (31%)	48 (35%)		11 (22%)	
Exclusive Formula	47 (19%)	42 (13%)	21 (15%)		7 (14%)	
Breastmilk and Formula only	40 (16%)	60 (19%)	15 (11%)		6 (12%)	
Other or Unknown	86 (35%)	114 (36%)	55 (40%)		27 (53%)	

Regarding maternal characteristics, increased maternal age was found to be significantly associated with higher parity (*p* < 0.0001), although mean ages were similar across all parities ranging from 32 to 34 years. Maternal ethnicity exhibited a significant association with parity (*p* < 0.0001), with a higher proportion of Hispanic or Latino mothers in higher parity groups and a higher proportion of non-Hispanic or Latino mothers in lower parity groups. Maternal race was also found to be significantly associated with parity (*p* = 0.0042). Caucasian women were more common in all parity groups with higher representation in lower parity groups of 1 and 2, while African American/Black, Asian, and other races showed varying distributions across the groups. Lower household income and education levels were also associated with high parities, Higher maternal pre-pregnancy BMI was also found to be significantly associated with higher parity (*p* = 0.0002) but not weight gain in pregnancy based on the American College of Obstetrics and Gynecology guidelines.^16^ Out of a total of 746 mothers, 295 (39.5%) delivered by Cesarean Section (CS), while 420 (56.3%) received peripartum antibiotics (ppabx, which is defined as the administration of systemic antibiotics within 48 h before delivery). Neither of these factors was found to be associated with parity. Of the mothers who had ppabx exposure, 295/420 (70.0%) had this exposure for CS prophylaxis, with cefazolin being the most frequently used antibiotic in this group (256/295, 86.8%). Additionally, 113/420 (26.9%) received ppabx for Group B Streptococcal prophylaxis, where penicillin was the most common antibiotic used (100/113, 88.5%). The remaining 12/420 (2.9%) had ppabx for a variety of reasons including chorioamnionitis and prolonged premature rupture of membranes.

In terms of infant characteristics, 381/746 (51.1%) of infants were males. Infant antibiotic use up to 6 months of life was found to be significantly associated with higher parity, although there were a large number of unknowns for this variable. Although there was a higher proportion of preterm births with higher parity, there was no difference in gestational age in weeks by parity. No significant associations were found between other infant clinical or demographic characteristics and parity, including infant sex and breast milk intake at 6 months.

### Microbiome differences by parity

16S rRNA gene sequencing of the V4 hypervariable region was conducted on 1647 serial stool samples from 746 infants collected around 2 months (m) (500 samples), 6 m (490 samples), 12 m (453 samples) and 24 m (204 samples) of life. After quality processing, the community matrix was composed of 3011 ASVs representing 639 unique species. Any demographic and clinical variables that were found to have an independent effect on the microbial composition were then checked to see if they had a confounding effect on the parity-microbial community interaction (R2 change > 10%, Supplemental Table S3). Confounders were found to be maternal ethnicity, ppabx and delivery mode and were adjusted for or explored accordingly.

**Figure 1.**
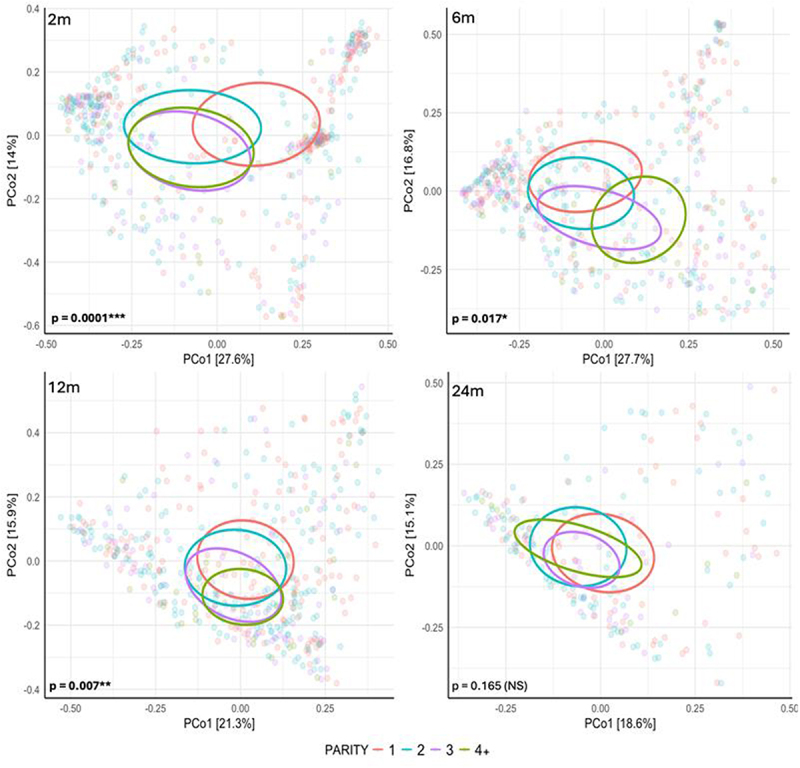
Principal coordinates analysis plots for each of the four time points, representing infant microbiome beta diversity (Bray-Curtis dissimilarity) based on parity groups. Ellipses represent 20% contours of sample distributions for each parity. PERMANOVA p-values based on parity, presented in lower left corner of each plot.

No differences were found with parity on infant microbiome alpha diversity. Beta diversity based on Bray-Curtis dissimilarity showed significantly different community compositions by parity (*p* < 0.05) in the first year of infant life, with parity-based community variation being highly significant at 2 months of life (*p* = 0.0001) and this significance disappearing by 24 months of life (Figure 1).

**Figure 2.**
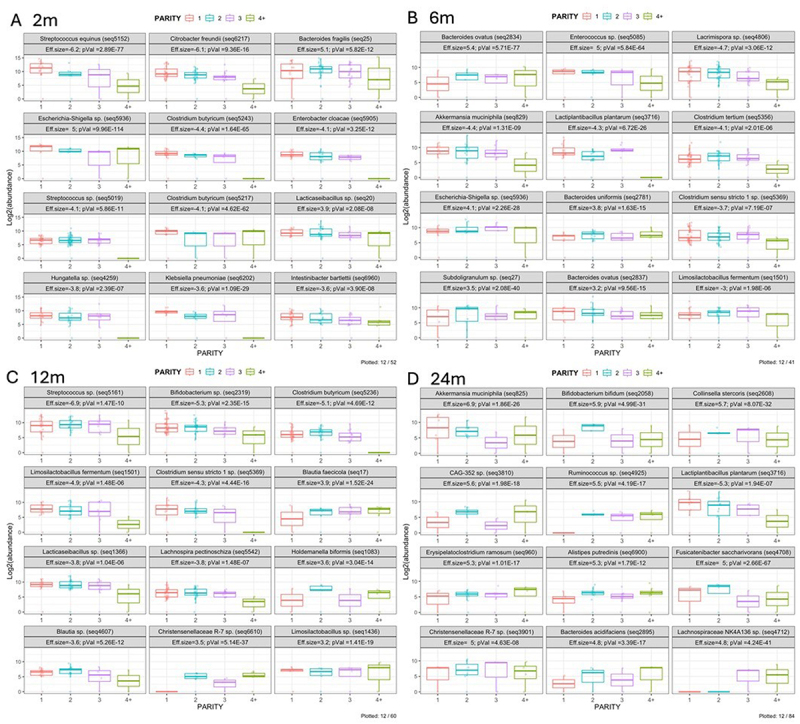
Box plots displaying the abundance distributions of top 12 most differential taxa (showing highest effect size and lowest p-values) across parity groups, for each timepoint. A: 2 months, B: 6 months, C: 12 months, D: 24 months.

The differential abundance of multiple taxa significantly differed by parity at each timepoint (Supplementary Table S1), with the 12 taxa demonstrating the biggest change in effect size by parity for each timepoint shown in Figure 2 (all p-values adjusted were by the Benjamini-Hochberg method for multiple comparisons). At 2 m, pathobiont species such as *Streptococcus equinus* and *Klebsiella pneumoniae*, as well as beneficial species such as *Clostridium butyricum* were decreased at higher parities (*p* < 0.001, Figure 2(A)). Similarly, at 6 m, *Enterococcus sp* and *Akkermansia muciniphila*, classified as a pathobiont and a beneficial species, respectively, were decreased at higher parities (*p* < 0.001, Figure 2(B)). At the 12 m timepoint, pathobiont species such as *Streptococcus sp* and beneficial species such as *Bifidobacterium sp* and *Clostridium butyricum* showed a decrease at higher parities (*p* < 0.001, Figure 2(C)). *Akkermansia muciniphila* was also decreased with increasing parity up to parity 3 at 24 m (*p* < 0.001, Figure 2(D)). Overall, different taxa varied by parity at different timepoints, with no consistent trends across timepoints. However, when comparing primiparous and multiparous (4+) groups, *Negativicutes spp*. (predominantly *Veillonella sp*) were more abundant in infants of primiparous mothers at all timepoints (Supplemental Figure S1).

### Modulation of microbiome differences by parity by other clinical factors

Maternal factors known to disrupt the maternal to infant microbiome transfer and infant microbiome composition, namely, delivery mode and ppabx, were then examined. Both delivery mode and ppabx were found to be significant contributors to infant microbiome composition at 2 m (*p* = 0.002 and 0.004 respectively, Supplemental Figure S2, Panel A). Given that the standard of care for CS delivery includes ppabx for post-operative infection prevention, delivery mode was adjusted for ppabx in our analysis. After adjustment, the effects of delivery mode were no longer significant at 2 m (*p* = 0.4, Supplemental Figure S2, Panel A). At all other timepoints, the effects of delivery mode were found to be nonsignificant on infant microbiome beta diversity, and this remained true even after adjusting for ppabx. Of note, ppabx alone had no significant effect on microbial community variation (beta diversity) after the first 2 months of life (Supplemental Figure S2, Panels B,C,D).

**Figure 3.**
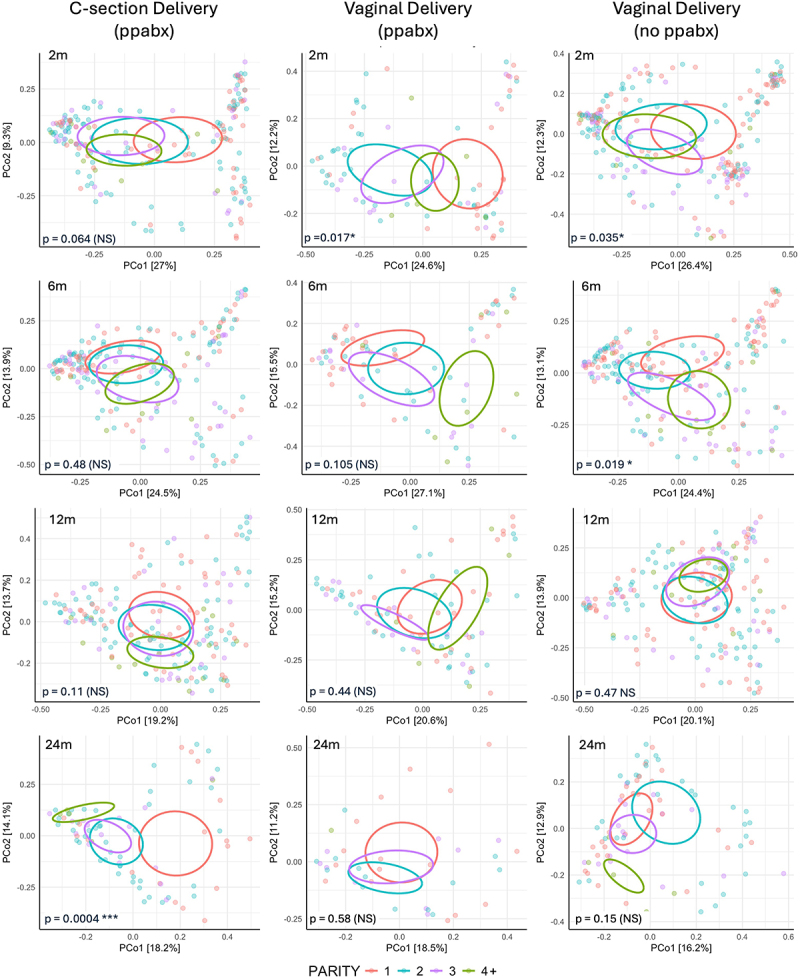
Principal coordinate plots for infant microbiomes (Bray-Curtis dissimilarity)) representing parity group differences for each type of delivery mode and peripartum antibiotic use (ppabx), and at each of the four time points. Ellipses represent 20% contours of sample distributions for each parity. PERMANOVA statistical test p-values for each subset, shown in lower left corner. P-values for Vaginal delivery adjusted for peripartum antibiotics dichotomy within vaginal delivery group (“adonis2(brayDist ~ delivMode + periABX + Ethn,” by = ‘margin,’ data = metadata)”).

After examining the individual effects of delivery mode and ppabx on the infant microbiome composition, we also analyzed the effect of parity within each mode of delivery and with ppabx use (Figure 3). In VD infants, both with and without ppabx, parity had a significant effect on community variation at 2 m (*p* = 0.035 and *p* = 0.017 respectively). The effect of parity remained significant in VD infants without ppabx to 6 months of age (*p* = 0.019), however lost significance by 6 months in those delivered by VD with ppabx. Conversely, in CS delivered infants, parity was not found to have a significant effect on beta diversity of the infant communities at any timepoint within the first year of life. Interestingly, parity did, however, show an effect on microbiome composition at 24 m in CS delivered infants (*p* = 0.0004).

## Discussion

In this brief report, we aimed to elucidate how maternal parity affects infant microbiome composition over time, as well as how maternal parity may interact with other maternal factors such as delivery mode. Overall, our results from this large maternal-infant longitudinal cohort demonstrated that maternal parity significantly affected the infant microbiome composition through the first year of life, with a stronger effect observed early in life. However, the effects of parity on microbiome composition were diminished with CS delivery.

### Maternal parity influences infant gut microbiome early in life

The infant gut microbiome composition was significantly associated with maternal parity through the first year of life, with the strongest signal produced at 2 months. Similarly in a recent longitudinal study examining sows and their piglets’ microbiomes, parity influenced microbiome composition most significantly at weaning (day 0), with diminishing effects over time up to day 42.^9^ Another study in a pig model found higher alpha diversity was seen in offspring born to primiparous mothers compared with those born to multiparous (parity of 7 or higher) mothers, with the difference decreasing over time. Together, these studies suggest a significant but transient effect of parity on the developing microbiome composition early in life. However, it has been shown that even a transient change in the infant gut microbiome during a critical window of gut microbiome development can influence later health^15^.

In our study, we demonstrated that as parity increased, the differential relative abundance of both potentially beneficial taxa and pathobionts decreased at each timepoint, although the specific taxa varied across timepoints (Figure 1(B)). Potentially beneficial taxa that decreased with parity included *Clostridium butyricum* and *Akkermansia muciniphila* at 2 and 12 m, which are producers of short-chain fatty acids necessary for regulating gut homeostasis^17–21^. Pathobionts that decreased with parity included *Klebsiella pneumoniae* at 2 m and *Streptococcus spp* at 2 and 12 m, which can cause opportunistic infections.^22^ More consistently, when comparing infants born to primiparous women and those with the highest parity (4+), *Veillonella spp*, previously associated both with protective functions and inflammation, were consistently increased in those born to primiparous women.^23–29^ Differing from our results, in a small cohort study Kennedy et al. found higher protective *Bifidobacterium sp* in infants of multiparous women compared to primiparous women in meconium and at 6 m whereas we found the opposite pattern of decreasing *Bifidobacterium sp* with increasing parity and only at the 12 m timepoint^11,12^. Potential reasons for the discrepancies between studies may include the timing of the sample collection, grouping of parities or infant diets which may vary between cohorts (breast milk vs formula or a mix of both). Additionally, bead beating was utilized in our study for homogenization during DNA extraction, which may impact the recovery of Gram positive bacteria such as *Bifidobacterium*.^30^

While we have now demonstrated clearly that maternal parity influences the human early life gut microbiome composition, the physiological reason for this phenomenon remains unclear. Kennedy et al. hypothesized that the difference in maternal and infant microbiome by parity may be due to the gut microbiome holding an “ecological memory” of previous pregnancies, which could help reduce maternal strain in future pregnancies, such that previous pregnancies may have a persistent impact on the maternal microbiome which is transferred to the infant during pregnancy and birth.^11,12^ We propose, complementary to prior studies, that with each pregnancy the maternal immune system undergoes repeated hormonal and inflammatory shifts that may not fully return to baseline. Early in pregnancy, the immune system shifts to an anti-inflammatory state to tolerate the fetus, and by the end it shifts to a pro-inflammatory state to support labor.^31–33^ These cumulative changes in the maternal immune system may be associated with changes in the maternal gut microbiome, that may also not return to baseline, with subsequent microbial transfer to the infant, thus affecting the infant’s very early life microbiome.^34,35^ While the mechanisms remain unclear – whether potentially due to ecological memory or immune-driven shifts or both – parity should be considered when studying maternal-infant microbiome dynamics.

### Delivery mode and peripartum antibiotic use, in conjunction with parity, significantly affects infant microbiome composition

Given that parity-based microbiome shifts were seen more clearly in the early life of infants, we hypothesize in agreement with Kennedy et al. that parity influences the maternal gut microbiome, which is reflected in the infant gut microbiome following maternal-infant transfer at birth.^11^ Of note, parity has also been shown to influence the maternal vaginal microbiome in addition to the maternal gut microbiome.^36^ To further examine this hypothesis, we performed a sub-analysis of parity by delivery mode and ppabx use, as both CS and ppabx use are known to be a major factors that interrupt maternal gut microbiome and vaginal microbiome transfer to the infant at birth and subsequent gut microbiome colonization.^37,38^ Our study revealed significant differences in microbiota composition between parity groups among VD infants at 2 months of age, with a longer lasting effect in VD infants who did not receive ppabx with significant changes lasting up to 6 months of age. However, no significant effects of parity were seen on the microbiome of CS delivered infants in the first year of life. This supports the hypothesis that early parity-related changes in the microbiome originate from the maternal microbiome which is transferred to an infant at birth in a VD but interrupted during a CS delivery. It is also well recognized that delivery mode-related differences in the microbiome composition start to converge in the first year of life, and indeed no parity-related differences in VD infants were seen after 6 m.^39^ Interestingly, a parity related difference in microbiome composition was observed in CS delivered infants at 24 months. We suspect that this might reflect indirect influences of parity – such as household composition, sibling microbiota sharing, or maternal behavior – that take longer to shape the microbiome in the absence of initial microbial seeding.^40^ Accordingly, Jokela et al. also showed that VD infants had parity-related microbiome composition changes from very early in life, whereas CS delivered infants only started to show parity-related changes later in infancy.^13^ Therefore, there is likely a dual mechanism effect of parity on the infant microbiome, with very early life parity-related changes likely due to maternal microbiome to infant transfer and changes later in infancy due to other parity-related factors such as siblings in the house and household composition.^13,40^

### Study limitations

Our study examines the effect of parity on the longitudinal infant gut microbiome over the first 2 years of life in a large cohort study. The major strengths of our study included its large sample size of 1,647 samples from 746 infants, longitudinal study design over a two-year period, and breadth of clinical information on both maternal and infant participants. In addition, our cohort had a wide range of parity groups, allowing for detailed analysis of the effects of parity on the infant microbiome. A limitation of our study was that it was a single center study with limited diversity and therefore may be less generalizable. Additionally, although we had a large sample size with various parity groups, 16S rRNA gene sequencing was used for microbiome analysis. Utilization of shotgun metagenomic sequencing may have provided deeper insight into characteristics of the microbiome at a species level, as well as non-bacterial microorganisms and their functional properties. While we hypothesize that the early effects of parity on the infant microbiome are due to the effects on the maternal microbiome and subsequent maternal to infant transfer, we did not have maternal samples to be able to confirm this. While it was believed later effects of parity on the microbiome may be due to siblings in the house, we had limited sibling and household composition data and no sibling samples to be able to assess this. Furthermore, it is known that breast feeding length varies by parity and so could be an important confounder, however we had limited breast feeding data available to be able to assess this.^41^ Lastly, there were limited samples available at parity 4+ at later timepoints, which may limit this analysis.

### Conclusions

Parity significantly affected early infant gut microbiome composition in the first year of life, with the clearest signal found in the first few months of life. However, the effects of parity were diminished with CS delivery, which we hypothesize is due to decreased mother-to-infant microbiome transfer with CS. While the underlying mechanism of parity’s effects on the microbiome and impact on infant health outcomes needs to be further explored, these results demonstrated the necessity of including parity as a variable to account for in longitudinal infant microbiome analyses.

## Materials and methods

### Study design and sample collection

#### Enrollment

Mothers were enrolled prenatally with informed consent in a longitudinal, prospective cohort study “The First 1000 Days of Life and Beyond” within the Inova Health System. All experimental protocols were approved by the Inova Health System and WCG Institutional Review Board (Inova protocol #15–1804, WCG protocol #20120204). In brief, serial stool samples were collected from infants at approximately 2 m, 6 m, 12 m, and 24 m of age as previously described.^42–44^ All samples were collected by caregivers at home and mailed back to the lab, using previously validated methods and stored at −80°C until analysis.^44^

Maternal demographic information including parity (parity after the delivery of the current infant from pregnancy in this study), mode of delivery (VD versus CS), use of prenatal antibiotics (antibiotics given to mother from conception to 2 days before delivery) and ppabx (antibiotics given to mother up to 2 days before and during delivery) was collected via questionnaires and electronic medical records review. In addition, maternal race/ethnicity, pre-pregnancy BMI, and maternal weight gain during pregnancy were collected through the same methods. Infant data such as gestational age at delivery, NICU admission, use of antibiotics, and medical history were also collected from parents through questionnaires.

#### DNA extraction and microbiome sequencing

DNA was extracted from stool aliquots using the DNeasy PowerSoil Pro kit (Qiagen, Valencia, CA) following manufacturer’s instructions. Bead beating was used for homogenization using the TissueLyser II (Qiagen, Valencia, CA). Barcoded PCR primers annealing to the V4 region of 16S ribosomal RNA gene were used for library generation (forward primer 5’-GTGYCAGCMGCC-GCGGTAA-3,’ reverse primer: 5’-GGACTACN-VGGGTWTCTAAT-3’). Sequencing was performed on the Miseq platform (Illumina, CA, USA) with 2 × 250 base pair paired end reads. Positive controls (DNA sequences) and negative controls (DNA-free water) were used.

#### Microbiome and statistical analysis

Sequence quality control and processing to an amplicon sequence variant (ASV)-based community composition matrix, were performed as described in prior studies^42^. Community differences were explored visually and statistically between these variables, independently for each time point, with the help of R packages “ggplot2,” “ampvis2,” “lmerTest,” “DESeq2,” “vegan” and “pairwiseAdonis.” Alpha diversity was explored using Shannon index and groups were compared statistically with ANOVA and Wilcoxon rank-sum testing. Beta diversity was explored using Bray-Curtis dissimilarity, PERMANOVA statistical testing and PCoA visualization. Variables tested for community response were categorized as demographic (maternal age, race, and ethnicity), socioeconomic (household income and education level), maternal clinical factors (delivery mode, prenatal antibiotics, ppabx, prepregnancy BMI, and gestational weight gain) and infant clinical factors (gestational age at birth, infant antibiotic use). Variables were also evaluated for confounding effect on microbial community response to parity. Variables were considered confounders when they displayed an independently significant effect on microbial community, in combination with influence on parity – microbial community interaction (a change in effect size of parity (R2 statistic) by more than 10%, when the variable is added to the PERMANOVA model). Confounders were adjusted for in all models evaluating the microbial response to parity (e.g. adonis2 (bray ~ confounder + parity, data = metadata)) and/or addressed through stratification. Variables that showed only effect on parity but no microbial community effect or no effect on microbial community response to parity, were not considered confounders. Infant diet variables (breastmilk, formula, solid food) had to be excluded from analysis, due to insufficient information likely producing unreliable results. Prior to differential abundance testing, additional community matrix filtering was performed to remove lowest prevalent ASVs for each time point ( < 2% across samples) to reduce false positives. Differential abundance testing on ASV level taxonomy was performed using DESeq2 or MaAsLin2 comparing across the different parities and p-values adjusted were by the Benjamini-Hochberg method for multiple comparisons. Correlation between clinical and demographic factors with parity groups was evaluated using the Chi-square test and ANOVA test for categorical and continues variables, respectively.

## Supplementary Material

SupFig2_revised.jpg

Supplemental Table 3.docx

Supplemental Table 1.xlsx

Supplemental Figure 1.jpg

Supplemental Table 2.docx

## Data Availability

The data that support the findings of this study are openly available in: **PRJNA1274022**.
