# Supplementary figures and images for: Parity influences on the infant gut microbiome development: a longitudinal cohort study
Source: Gut Microbes. 2025 Sep 9;17(1):2557980. doi: 10.1080/19490976.2025.2557980 (PMC12427479; doi:10.1080/19490976.2025.2557980)

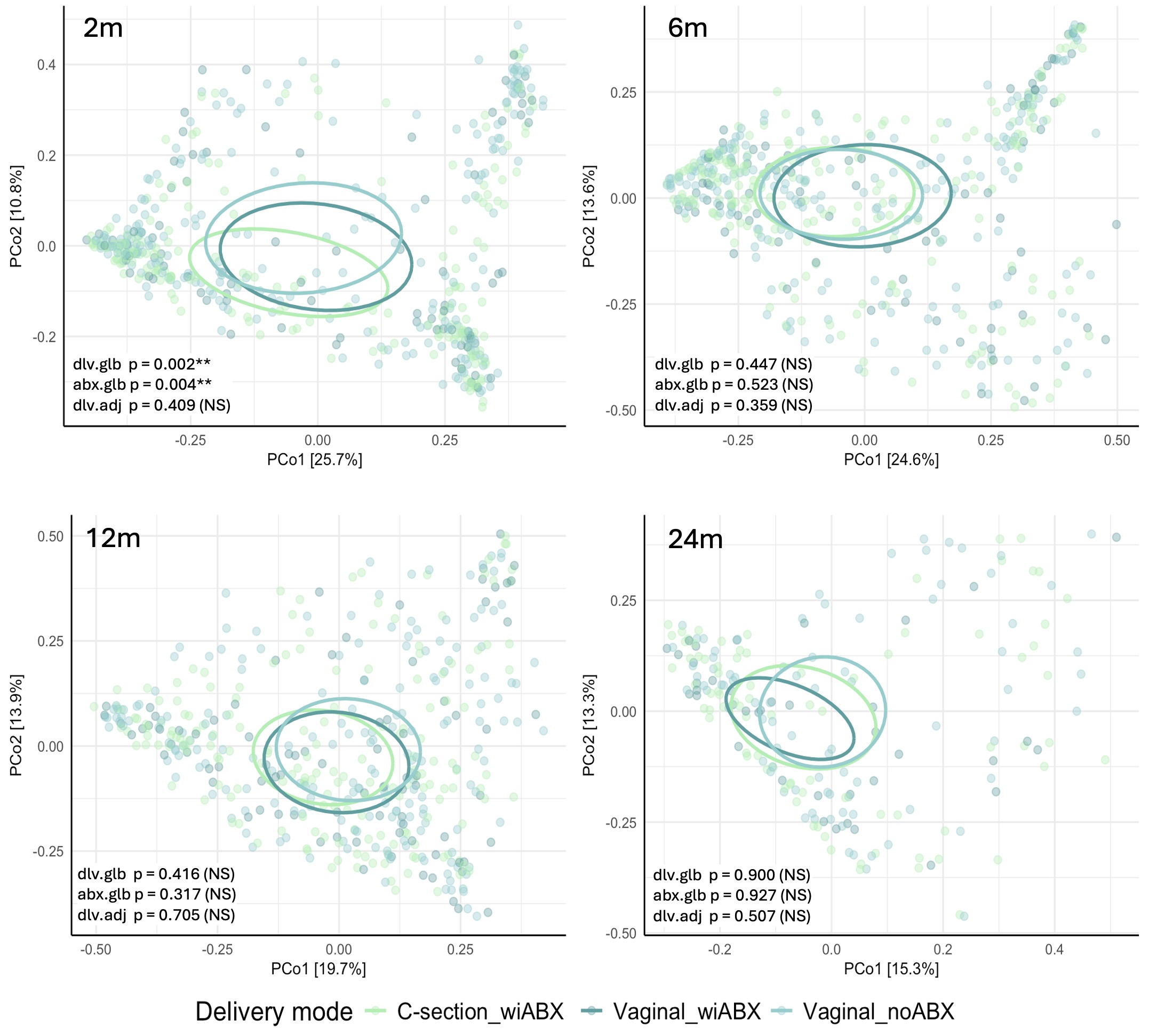

Supplement: SupFig2_revised.jpg [file KGMI_A_2557980_SM1848.jpg]

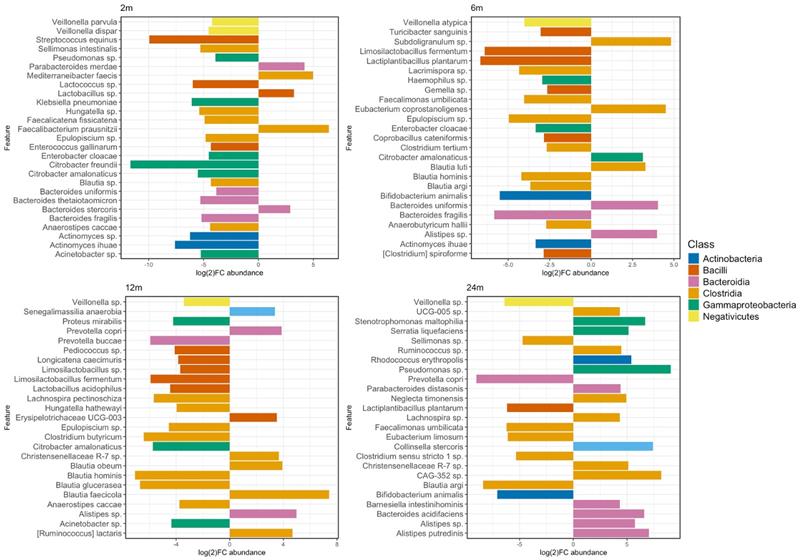

Supplement: Supplemental Figure 1.jpg [file KGMI_A_2557980_SM1845.jpg]
