# Supplementary material for: Parity influences on the infant gut microbiome development: a longitudinal cohort study
Source: Gut Microbes. 2025 Sep 9;17(1):2557980. doi: 10.1080/19490976.2025.2557980 (PMC12427479; doi:10.1080/19490976.2025.2557980)
Supplement: Supplemental Table 3.docx [file KGMI_A_2557980_SM1847.docx]

**Supplemental Table 2**

| **Main variable** | **Test variable** | **Test for** | **R2p** |
| --- | --- | --- | --- |
| Maternal parity |  | Self | 0.89 |
| Maternal parity | Maternal Age | Conf | 0.87 |
| Maternal parity | Maternal ppabx | Conf | 0.89 |
| Maternal parity | Maternal Ethnicity | Conf | **0.74** |
| Maternal parity | Delivery mode | Conf | 0.88 |
| Maternal parity | Maternal pregestational BMI | Conf | 0.86 |
| Maternal parity | Maternal gestational weight gain | Conf | 0.88 |
| Maternal parity | Household income | Conf | 0.85 |
| Maternal parity | Maternal education | Conf | 0.79 |
