# Supplementary material for: Parity influences on the infant gut microbiome development: a longitudinal cohort study
Source: Gut Microbes. 2025 Sep 9;17(1):2557980. doi: 10.1080/19490976.2025.2557980 (PMC12427479; doi:10.1080/19490976.2025.2557980)
Supplement: Supplemental Table 2.docx [file KGMI_A_2557980_SM1844.docx]

**Supplemental Table 2**

|  | **Parity** | | | |
| --- | --- | --- | --- | --- |
|  | **1** | **2** | **3** | **4** |
| 2m | 164 | 214 | 95 | 27 |
| 6m | 165 | 202 | 92 | 31 |
| 12m | 148 | 194 | 79 | 32 |
| 24m | 62 | 90 | 41 | 11 |

Number of samples at each timepoint for analysis in Figure 1, Figure 2, Supp Fig 1

|  | **Parity** | | | | | | | | | | | |
| --- | --- | --- | --- | --- | --- | --- | --- | --- | --- | --- | --- | --- |
|  | **1** | | | **2** | | | **3** | | | **4** | | |
|  | CS | VD with abx | VD w/o abx | CS | VD with abx | VD w/o abx | CS | VD with abx | VD w/o abx | CS | VD with abx | VD w/o abx |
| 2m | 57 | 26 | 81 | 90 | 38 | 86 | 33 | 15 | 47 | 13 | 5 | 9 |
| 6m | 60 | 28 | 77 | 91 | 29 | 82 | 40 | 14 | 38 | 11 | 7 | 13 |
| 12m | 53 | 29 | 66 | 82 | 36 | 76 | 27 | 15 | 37 | 16 | 5 | 11 |
| 24m | 18 | 16 | 28 | 44 | 13 | 33 | 15 | 9 | 17 | 5 | 1 | 5 |

Number of samples at each timepoint for analysis in Figure 3. CS= Cesarean Section, VD = vaginal delivery, w/o = without.
